# Paravertebral extraskeletal myxoid chondrosarcoma: a case report and review of the literature

**DOI:** 10.11604/pamj.2015.21.213.6639

**Published:** 2015-07-23

**Authors:** Fatima Zahra Farhane, Zineb Alami, Touria Bouhafa, Abderrahmane Elmazghi, Khalid Hassouni

**Affiliations:** 1 Department of Radiotherapy, Hassan II University Hospital, Fez, Morocco

**Keywords:** Extraskleletal myxoid chondrosarcoma, paravertebral, malignant tumour

## Abstract

The extraskeletal myxoid chondrosarcoma (CME) is a rare malignant soft tissue tumour described as a distinct clinical, histological, immunohistochemical, genetical and evolutive entity. It represents only 2.5% of soft tissue sarcomas. Its individualization is important because it has a long and indolent clinical course, and tumour-related death often occurs after a long survival period. The diagnostic key is morphological supported by immunohistochemistry and genetics t (9; 22) that allow differentiating it from other tumours with myxoid stroma and from chordoma. This report describes a patient with paravertebral extraskeletal myxoid chondrosrcoma with a high locoregional extension.

## Introduction

Extraskeletal chondrosarcomas were first described by Stout and Verner in 1953 [1]; however, it was not until 1972 that extraskeletal myxoid chondrosarcoma (EMC) was histopathologically defined as its own entity [2]. EMC is provisionally classified as a tumor of uncertain differentiation in the revised version of the World Health Organization classification of tumors of soft tissue and bone in 2002 [3]. EMC is a relatively rare but well- characterized tumor [4]. We report a case of paraspinal location with a high locoregional extension.

## Patient and observation

**Figure 1:**
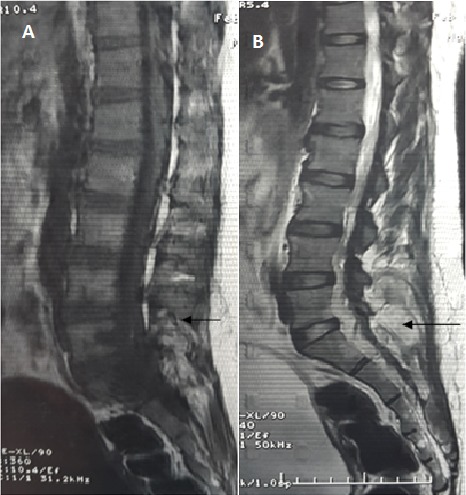
Preoperative MRI shows lesion with low signal in T1WI and heterogeneous high signal in T2WI

**Figure 2:**
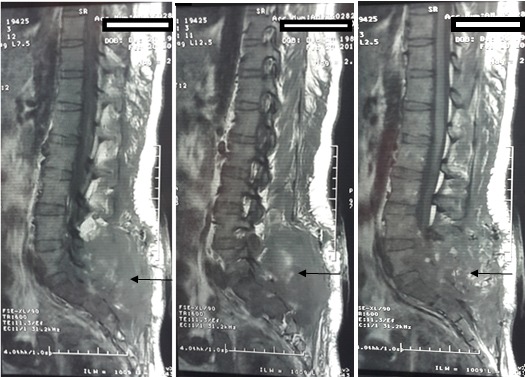
Postperative MRI shows lesion with low signal in T1WI and heterogeneous high signal in T2WI

A 25-year-old female presenting with 3 months history of cauda equina syndrome progressively worsening. Lumbar spine medical imaging revealed in MRI scan a low signal intensity process on T1 a high signal intensity on T2 (Figure 1) and in CT scan a hypodense process extending on a height of 7cm and developing in the left posterior lumbar soft tissues at the height of L5 extending in the left sacral region with infiltration and osteolysis of the sacroiliac joint and the left sacral foramina with nerve root compression at this level. The patient underwent surgery with resection of a left paravertebral lesion + L5 and S1 laminectomy + partial resection of the intraductal portion of the tumor. Histopathological study showed that the tumor is characterized by multilobular structures divided by fibrous septa, and disposed in an abundant myxoid matrix. tumoral cells are isolated or grouped in cords, mitoses are rare. Cells express the protein S100 and NSE and do not express cytokeratin AE1 / AE3 and desmin. Based on these findings, the patient was diagnosed with EMC. Postoperative Spinal MRI showed the persistence of a large tumor mass of the erector muscles of the lumbar spine 68x12mm extending to the gluteal muscles, with persistence of the anterior epidural tumoral component and extension at lumbosacral spinal (Figure 2). At his admission to the department of radiotherapy, 4 weeks later, clinical examination found a huge mass in the left buttock and posterior lumbar region. Planning CT and CT scanner found an increase in tumor volume with the same extensions described in the postoperative imaging in addition to extension at the left iliac bone and at the left iliac psoas muscle protruding at the left lateral pelvic region. From these results, a palliative radiotherapy was delivered in the total dose of 20 Gy in 5 fractions of 4Gy. The patient was referred for chemotherapy thereafter.

## Discussion

The CME is a rare entity, distinct clinically, histologically, immunohistochemistry, cytogenetics and scalable. It represents 2.5% of soft tissue sarcomas [5]. Approximately 80% of these tumors occur in the extremities, with 20% located in the trunk. The lower extremity is the most common location of EMC [4]. The male to female ratio of EMC is 2:1, with a peak occurrence in the fifth and sixth decades [4]. EMC is a relatively rare neoplasm with no specific findings in the clinic. Patients commonly present with non-specific symptoms, including tenderness and the detection of a palpable mass [3]. The most common manifestation of EMC is an enlarging soft tissue mass; some lesions are accompanied by pain and tenderness, or may restrict the range of motion. Radiological images are not specific; the lesions exhibit low density on CT, low signal intensity on T1-weighted MRI scans and high signal intensity on T2-weighted MRI scans [6]. The tumor ranges from approximately 1-25 cm (mostly 6-13 cm) in size and usually has a multilobular or nodular configuration with a relatively well-defined margin and an incomplete fibrous capsule [2, 5, 7–12]. The cut surface is gray to tan-brown and shows a gelatinous appearance, often accompanied by intralesional hemorrhage. The tumor is characterized by multilobular structures divided by fibrous septa of variable thickness, which is a consistent morphological configuration of EMC. Each lobule is typically composed of a proliferation of short spindle or oval cells arranged in clusters or short anastomosing cords or strands, often displaying a lacelike appearance and embedded in an abundant myxoid matrix. The tumor cells are generally small and have hyperchromatic or vesicular nuclei and eosinophilic or sometimes vacuolated cytoplasm. These features are somewhat reminiscent of chondroid tissues or lesions. However, the tumor usually lacks discernible cartilaginous histology. Mitotic figures are rare in most cases [5].

Immunohistochemically, the cells express vimentin sometimes SNP, NSE and PS100 [11, 5]. Cytokeratin and EMA are sometimes expressed focal manner [11]. Positivity of GFAP was reported. In electron microscopy, the presence of parallel microtubules and abundant REG are quite characteristic but nonspecific [9, 13]. Certain studies have shown that they may also be positive for Leu-7 and epithelial membrane antigen. Uniformly, they are negative for keratin, SMA and desmin [14–16] Genetically, CME is associated in about 80% to recurrent specific chromosome translocation t (9; 22) (q22; q12), leading to a genetic rearrangement between the EWS gene, located on chromosome 22 and the gene TEC, located on chromosome 9 [11]. The rearrangement can be searched by RT-PCR on fixed tissue. Other translocations are rarely observed [5]: t (9; 15) (q22; q12), t (9; 17) (q22; q11.2), t (9; 17; 15) (q22; q11; q22), t (2; 13) (q32; p12) and t (11; 22) (q11; p11) [17].

EMCS should be distinguished from a variety of myxoid or cartilage-forming, benign, or malignant, and soft tissue or bone tumors. For example, intramuscular myxoma, nerve sheath myxoma/neurothekeoma, extraskeletal chondroma, myoepithelioma (mixed tumor) of soft parts, parachordoma, metastatic adenocarcioma, myxoid liposarcoma, low-grade myxofibrosarcoma/myxoid malignant fibrous histiocytoma, epithelioid sarcoma, malignant peripheral nerve sheath tumor, and extraskeletal or skeletal chondrosarcoma with prominent myxoid change. Among them, parachordoma and myoepithelioma of soft parts are occasionally difficult to differentiate from EMCS. Recognition of the histologic features of conventional EMCS that are present, at least in part, is most important for a differential diagnosis. Ancillary approaches such as immunohistochemistry and electron microscopy are also valuable [5]. Treatment is firstly surgery of the initial tumor and / or metastasis, radiotherapy and chemotherapy as first-line have not been proven [18]. This tumor does not respond to chemotherapy and the results concerning radiotherapy are discordant [19].

The development of CMES is difficult to predict. Nearly half of patients have one or more local recurrences within an average of 44 months [20]. Survival after the onset of metastases is very varied in duration, on average 66 months. Although the five-year survival rate is quite high, metastases are common (46-90% of cases) and long-term prognosis is adverse (30-70% survival at ten years) [20, 21]. Metastases occur in order of decreasing frequency, in lungs, soft tissue, lymph nodes, bone, and brain. The main poor prognostic factors are the occurrence in man, the late age of onset, tumor size greater than 10 cm, proximal seat, the incompleteness of the initial surgical resection, the absence of surgical resection and discovery of metastases at diagnosis [11, 20]. The histoprognostic criteria such as necrosis, mitotic activity, the degree of differentiation does not appear to influence the outcome [22]. In fact, these criteria are discordant in the literature and are controversial subjects.

## Conclusion

The CME is a rare entity, distinct clinically, histologically, immunohistochemistry, cytogenetics and scalable. The diagnostic key is morphological, aided by immunohistochemistry and genetic study.
